# Neuroimaging Investigations of Obesity: a Review of the Treatment of Sex from 2010

**DOI:** 10.1007/s13679-023-00498-0

**Published:** 2023-03-18

**Authors:** Lisa A. Kilpatrick, Hyeon Min An, Shrey Pawar, Riya Sood, Arpana Gupta

**Affiliations:** 1 Vatche and Tamar Manoukian Division of Digestive Diseases, David Geffen School of Medicine, University of California, Los Angeles, USA; 2 David Geffen School of Medicine, Goodman-Luskin Microbiome Center, University of California, Los Angeles, USA; 3 G. Oppenheimer Center for Neurobiology of Stress and Resilience, The Obesity and Ingestive Behavior Program, Vatche and Tamar Manoukian Division of Digestive Diseases, David Geffen School of Medicine, University of California, 10833 Le Conte Avenue, Center for Health Sciences 42-210, Los Angeles, CA 90095 USA

**Keywords:** Sex differences, Obesity, Multimodal neuroimaging, Co-occurrence analysis, Affect, Motor

## Abstract

**Purpose of Review:**

To summarize the results of adult obesity neuroimaging studies (structural, resting-state, task-based, diffusion tensor imaging) published from 2010, with a focus on the treatment of sex as an important biological variable in the analysis, and identify gaps in sex difference research.

**Recent Findings:**

Neuroimaging studies have shown obesity-related changes in brain structure, function, and connectivity. However, relevant factors such as sex are often not considered.

**Summary:**

We conducted a systematic review and keyword co-occurrence analysis. Literature searches identified 6281 articles, of which 199 met inclusion criteria. Among these, only 26 (13%) considered sex as an important variable in the analysis, directly comparing the sexes (*n* = 10; 5%) or providing single-sex/disaggregated data (*n* = 16, 8%); the remaining studies controlled for sex (*n* = 120, 60%) or did not consider sex in the analysis (*n* = 53, 27%). Synthesizing sex-based results, obesity-related parameters (e.g., body mass index, waist circumference, obese status) may be generally associated with more robust morphological alterations in men and more robust structural connectivity alterations in women. Additionally, women with obesity generally expressed increased reactivity in affect-related regions, while men with obesity generally expressed increased reactivity in motor-related regions; this was especially true under a fed state. The keyword co-occurrence analysis indicated that sex difference research was especially lacking in intervention studies. Thus, although sex differences in the brain associated with obesity are known to exist, a large proportion of the literature informing the research and treatment strategies of today has not specifically examined sex effects, which is needed to optimize treatment.

**Supplementary Information:**

The online version contains supplementary material available at 10.1007/s13679-023-00498-0.

## Introduction

Obesity is a growing concern worldwide and impacts the quality of life. In the USA, the prevalence of obesity among adults was 42.4% in 2017–2018 [1]. Obesity is associated with major health risks, such as high blood pressure, high cholesterol, type II diabetes, arthritis, asthma, and poor overall health [2]. However, there are differences in health risks and treatment outcomes between men and women with obesity [3]. For example, in a meta-analysis, men had a greater risk for mortality with increasing body mass index (BMI) compared to that for women (hazard ratio: 1.51 vs 1.30 per 5 kg/m^2^, *p* < 0.00001) [4]. Examining sex differences in obesity provides important information that can be used to inform treatment regimens tailored to each sex. However, more research is needed to understand the biological basis for the influence of sex in obesity [3].

Numerous neuroimaging studies have shown associations between obesity and measures of various brain features, such as morphology, resting-state activity and connectivity, white matter microstructure, and task activation. However, the effects may vary according to sex. Although research has confirmed the involvement of the brain in obesity, a comprehensive multimodal summary of the effects of both obesity and sex on the brain is lacking. Therefore, this review aimed to summarize the results of adult obesity neuroimaging studies published from 2010, with a focus on the treatment of sex in the analysis. Specifically, studies were categorized according to whether the analysis comprised direct comparisons between men and women (direct sex); used data from only male or only female participants or separately analyzed male and female participants, providing disaggregated data and allowing qualitative comparisons (single/within sex); controlled for sex, disallowing comparisons (sex adjusted); or did not consider sex, disallowing comparisons (no sex). We considered the first two categories to adequately treat sex as an important biological variable in the analyses. Additionally, we performed a keyword co-occurrence analysis of the selected articles, as a snapshot of popular topics/concepts in the field of adult obesity neuroimaging their relationship to the topic of sex differences.

## Materials and Methods

### Database Search Strategy and Results

We searched three online electronic databases, PubMed, Embase, and Web of Science, for obesity neuroimaging studies published from 2010 to the present. This search was conducted using a predetermined set of words related to obesity, neuroimaging, and sex/gender. The search terms, selected in collaboration with a research librarian, are indicated in Supplemental Table S1.

### Eligibility Screening

Two stages of screening with predetermined inclusion and exclusion criteria were performed. The authors were contacted as needed. All studies considered for eligibility were published in a peer-reviewed journal.

In the first stage, we examined the title and abstract of each article for the following criteria: (1) investigation of obesity, (2) adult human participants, and (3) reporting of brain MRI data. Reasons for exclusion were as follows: (1) used non-human models; (2) included infant, child, or adolescent participants; (3) imaged non-brain tissues; (4) no structural magnetic resonance imaging (MRI), resting-state MRI (rsMRI), task/stimulus functional MRI (fMRI), or diffusion tensor imaging (DTI) was performed; (5) no measure of obesity was reported; (6) in treatment studies, no pre-treatment MRI data was reported; (7) investigated patients with anorexia nervosa or other eating disorders; (8) review article/protocol study/abstract only; and (9) duplicate article that was not previously identified.

In the second screening stage, we read the entire article, paying particular attention to the methods, results, and discussion sections. Articles that were found to violate the abovementioned study criteria upon full-text review were excluded.

### Data Extraction

After screening, we extracted data on the treatment of sex in the analyses, participant characteristics (sex, population), modality(s) used, and main findings. Articles were categorized according to how the researchers analyzed sex. The first category was “direct sex,” which was applied to studies that directly compared men and women in the analysis. The second category was “single/within sex,” which was applied to studies that included only male or only female participants or separately reported results for male and female participants, without a direct comparison, but allowing qualitative comparisons. The third category was “sex adjusted,” which was applied to studies that controlled for sex differences in the analysis, disallowing comparisons between the sexes. The fourth category was “no sex,” which was applied to studies that did not consider sex in the analysis (at most, the numbers of male and female participants were reported), disallowing comparisons between the sexes. The first two categories were considered to adequately treat sex as an important biological variable in the analyses. Subsequently, we organized the findings by the type of brain imaging performed, namely, DTI, structural MRI, rsMRI, and task/stimulus fMRI, with multimodal studies represented in multiple sections.

### Keyword Co-occurrence Analysis

A keyword co-occurrence network analysis of the included articles was conducted to examine popular topics/concepts in the field of adult obesity neuroimaging linked and not linked to the topic of sex differences. Keywords comprised those assigned to the article by the authors and/or the database (via various algorithms), with preference given to the keywords assigned by the Web of Science database for articles in multiple databases. Keyword co-occurrence networks represent each keyword as a node and each co-occurrence of a pair of words as a link, with the number of times that a pair of words co-occurs in multiple articles as the weight of the link connecting the pair. Keyword co-occurrence networks can be constructed to visualize the cumulative knowledge of a research area, aiding in uncovering meaningful knowledge and insights based on the patterns and strength of links between keywords that appear in the literature [5]. The citation records of the included articles were analyzed in VOSviewer (https://vosviewer.com) using LinLog normalization and modularity clustering techniques [6]. Other settings used in VOSviewer were as follows: counting method, fractional; attraction, 1; repulsion, 0; resolution, 1.1; minimum cluster size, 6. The top 100 keywords, based on the total strength of the co-occurrence links with other keywords (i.e., keywords frequently co-assigned to adult obesity neuroimaging articles), were selected.

## Results

### Study Selection

**Fig. 1:**
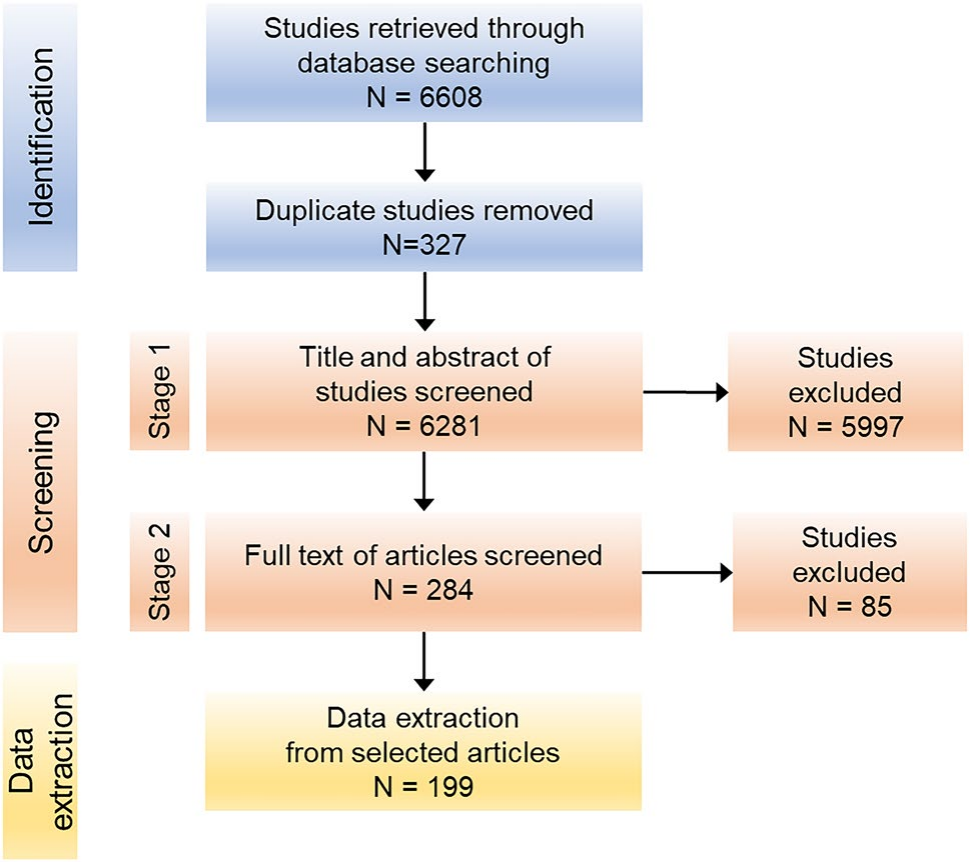
PRISMA flow diagram for the systematic review detailing the database search, number of studies screened, and the full texts retrieved

The PRISMA diagram illustrating the study selection process is shown in Fig. 1. In total, 190, 4982, and 1436 articles were retrieved from the PubMed, Embase, and Web of Science databases, respectively. These articles were imported into Endnote, and duplicates (*n* = 327) were removed. Thus, 6281 articles remained for further screening. After stage 1 screening, 5997 articles were excluded, and 284 articles were selected for full-text screening.

**Fig. 2:**
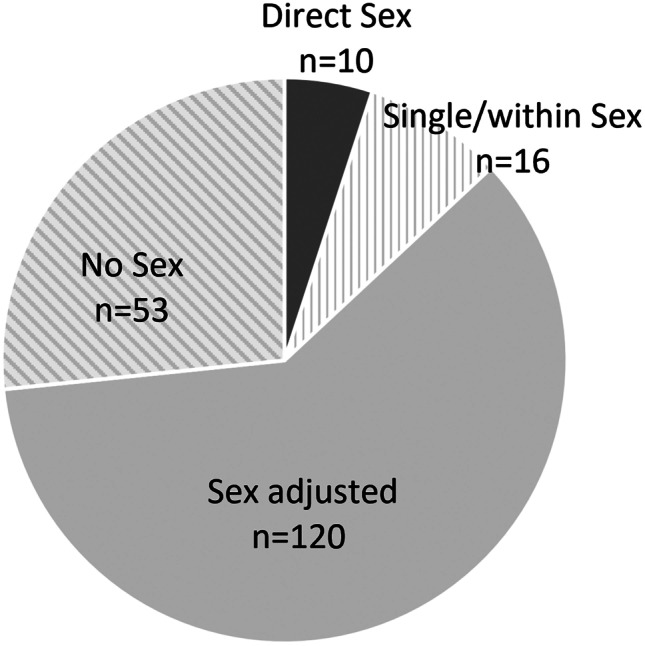
Pie chart showing the numbers and relative proportions of article types according to the treatment of sex in the analysis. Direct sex: the analysis comprised direct comparisons between men and women; single/within sex: the analysis used data from only male or only female participants or separately analyzed male and female participants; sex adjusted, the analysis controlled for sex; no sex: the analysis did not consider sex

After second-stage screening, an additional 85 articles were excluded. The reasons for exclusion are indicated in Supplemental Table S2. The remaining 199 articles were categorized according to the treatment of sex in the analysis. Ten studies (5%) were categorized as direct sex, 16 studies (8%) as single/within sex, 120 studies (60%) as sex adjusted, and 53 studies (27%) as no sex (Fig. 2). The full bibliography according to article type is provided in the Supplemental Materials.

### Structural MRI

#### Direct-Sex and Single/Within-Sex Studies

In total, 12 studies reported on sex differences/commonalities and single/within-sex obesity-related alterations in regional and total gray matter volume (GMV), regional white matter volume (WMV), and cortical thickness. Although most of these studies reported associations between obesity (based on BMI cut-off points), or higher values in measures of body size (e.g., BMI, waist-to-hip ratio) or total body fat, and lower GMV in both sexes, the spatial extent of the alterations may be more widespread in men than in women [7–9], suggesting sex differences in the robustness or severity of morphological alterations. For example, one study found negative associations between total body fat and subcortical GMV in the thalamus, caudate nucleus, putamen, globus pallidus, hippocampus, and nucleus accumbens in men, while only the globus pallidus was affected in women [10]. Furthermore, an association between obesity and increased pituitary gland volume only reached statistical significance in men [11]. Similarly, relationships between obesity/larger waist-to-hip ratio and cortical thickness in frontal, temporal, and occipital cortices were found in men, while no such relationships were found in women [12, 13].

Among patients with cerebral small vessel disease, reductions in hippocampal volume over a 9-year period were more prominent in men than in women with larger baseline waist circumference [8]. In contrast, another study reported that reductions in hippocampal volumes were more severe in women than in men with type II diabetes (T2DM) and obesity [14]. Furthermore, a study on structural brain networks (based on cortical thickness and GMV) found that visceral adipose tissue volume exacerbated the effect of aging on network covariance in both men and women, which was modulated by the estradiol level in women but not men [15].

A female-specific study found a positive association between high BMI and GMV in the left nucleus accumbens [16], unlike the abovementioned study conducted in patients with T2DM [14]. Another female-specific study found associations between BMI and decreased GMV in the left orbitofrontal (associated with worse executive function), right inferior frontal, right precentral gyri, parahippocampal, fusiform, and lingual gyri, and right cerebellar regions, as well as increased WMV in the frontal, temporal, and parietal lobes in older women [17]. A male-specific study found a relationship between total brain volume and higher resting energy expenditure (protective against obesity) in Malaysian men, but not in Chinese and Asian-Indian men living in Singapore [18].

#### Sex-Adjusted Studies

Sex-covariate studies from 2010 to present (*n* = 48) reported on obesity-related alterations in morphological parameters, as well as changes before/after intervention. Similar to the direct-sex and single/within-sex studies, sex-adjusted studies generally support obesity/high-BMI/larger waist circumference as related to lower total GMV [19–26]; however, this may be an over-simplification, given the abovementioned findings regarding a sex difference in the spatial extent of GMV alterations. Specific regions with obesity-related (e.g., high BMI, abdominal obesity, obesity-prone status) GMV reduction include the thalamus and hippocampus [22, 27–30],  which may have more robust obesity-related GMV reduction in men, based on the above-mentioned direct-sex and single/within-sex findings [10]. Additionally, cortical thickness alterations have been reported, with an overall thinner cortex and reduced regional cortical thickness in the ACC and posterior parietal cortices associated with obesity/higher BMI/larger waist circumference [31, 32]; these regions differ from those reported in direct-sex and single/within-sex studies [12, 13].

#### No-Sex Studies

No-sex structural studies from 2010 (*n* = 11) largely focused on the impact of bariatric surgery and other interventions on morphological parameters. These studies generally suggest that interventions can at least partially ameliorate obesity-related effects on these parameters [33–42].

### rsMRI

#### Direct-Sex and Single/Within-Sex Studies

Direct-sex and single/within-sex rsMRI studies (*n* = 4) reported on regional alterations in functional centrality (a graph theory parameter) and the amplitude and frequency distribution of spontaneous fluctuations. A graph theory study reported that increased early life adversity (ELA) was associated with increased centrality in reward and emotion regulation regions in women with high BMI and in somatosensory regions in men with high BMI [43]. Another study found associations between BMI and altered spontaneous fluctuation frequency distribution in subcortical reward-related regions, including the left globus pallidus and substantia nigra, in both men and women, and additional alterations in the right globus pallidus and bilateral putamen in women; furthermore, BMI was associated with reduced connectivity between left globus pallidus/putamen and emotion and cortical regulation regions in women but not men [44].

Additionally, a female-specific study found that high BMI was associated with altered frequency distribution in the nucleus accumbens, ACC, and ventral PFC/orbitofrontal cortex [16]. Another female-specific study found an association between obesity and increased flow-frequency activity in the putamen, claustrum, and insula under fasting and fed states [45].

#### Sex-Adjusted Studies

Sex-adjusted rsMRI studies (*n* = 13) have mostly reported on alterations in intrinsic connectivity between regions or within a network, not centrality or frequency distribution changes, rendering it difficult to compare the results to those in direct-sex and single/within-sex studies. However, several studies reported obesity-related increases in intrinsic connectivity or involving regions with increased centrality or altered frequency distribution in direct-sex and single/within-sex studies, including the ventral striatum, amygdala, and insula [46–49].

#### No-Sex Studies

No-sex resting-state studies (*n* = 13) largely reported on the impact of interventions on rsMRI parameters [40, 41, 50–54].

### fMRI Task/Stimulus

#### Direct-Sex and Single/Sithin-Sex Studies

Direct-sex and single/within-sex task-fMRI studies (*n* = 9) investigated obesity-related responses to high and low energy–dense foods, under fed and fasting conditions. In a fed state, men with obesity showed greater connectivity between the amygdala and subgenual ACC and between the ventral striatum and bilateral supplementary and primary motor areas, left postcentral gyrus, and left precuneus, and greater activation in bilateral supplementary motor areas (motor control), while women with obesity showed greater connectivity between the amygdala and the left angular gyrus and primary motor areas, and greater activation in the dorsal ACC, in response to high energy–dense food cues [55, 56]. In contrast, in a fasted state, men with obesity showed greater connectivity between the amygdala and bilateral supplementary frontal and primary motor areas, left precuneus, and right cuneus, and greater activation in a visual perception region (temporal-occipital cortex), while women with obesity showed greater connectivity between the amygdala and the inferior frontal gyrus and dorsomedial PFC, and greater/more robust activation in affective and reward-related regions (amygdala, insula, medial orbitofrontal cortex, etc.), in response to high energy–dense food cues [55–58]. Another study did not find any sex-based differences in the response to visual food cues (hedonic foods, neutral foods, and non-food objects) in the fed state; however, in the fasted state, there was higher activation in the nucleus accumbens and insula in women than in men with obesity [59]. In response to nutrition information graphics in a fasted state, both men and women with high BMI showed increased ACC activation compared to that in same-sex controls; additionally, women with high BMI showed increased insula activation and men with high BMI showed decreased PFC activation [60].

Female-specific studies reported associations between higher BMI and greater activation in reward and hedonic brain regions in response to visual food cues, under both fasted and fed states [61], in line with reduced neural responses to satiety [62]. A male-specific study showed that oxytocin administration attenuated functional connectivity between the ventral tegmental area (dopaminergic reward system) and the insula somatosensory cortex, amygdala, hippocampus, operculum, and middle temporal gyrus when viewing high-calorie foods [63].

#### Sex-Adjusted Studies

Sex-adjusted task-fMRI studies (*n* = 42) investigated responses to a wider range of stimuli (food and odor cues) and tasks (e.g., paradigms that focused on reward and executive function tasks) than direct-sex and single/within-sex studies. Studies using visual food-cue stimuli found greater obesity-related reactivity in the amygdala, insula, hippocampus, striatum, hypothalamus, ventral posterior cingulate, angular gyrus, putamen, and frontal cortex, including the dorsolateral PFC (in a fasting state) [64–67]; some of these regions show greater obesity-related reactivity in women based on the abovementioned direct-sex and single/within-sex findings [55–58].

#### No-Sex Studies

No-sex task-fMRI studies (*n* = 28) largely reported on the impact of interventions on reactivity to food-related cues [39, 68–85].

### Structural Connectivity (DTI)

#### Direct-Sex and Single/Within-Sex Studies

Direct-sex and single/within-sex DTI studies (*n* = 3) reported on common and sex-specific associations between BMI/body fat and DTI measures of fractional anisotropy (FA), mean diffusivity (MD), and axial diffusivity (AD), as well as structural centrality. Specifically, high BMI/total body fat was associated with decreased AD in the corpus callosum [86] and increased global FA [10] in both men and women, while higher total body fat was negatively associated with global MD in women only [10]. A graph theory study reported greater structural centrality in the amygdala, hippocampus, and nucleus accumbens and anterior mid-cingulate cortex regions in women than in men with high BMI, while high BMI was associated with greater structural centrality in putamen and posterior insula regions in both men and women [87].

#### Sex-Adjusted Studies

Sex-adjusted studies (*n* = 23) mainly reported associations between obesity/high BMI/adiposity and FA. One study reported high BMI as related to a reduction in the global FA [88], opposite of that found in an abovementioned direct-sex and single/within-sex study [10]. Other studies reported obesity-related reductions in FA in widespread regions, including the bilateral frontal corticospinal tracts, right brainstem, uncinate fasciculus, corpus callosum, cerebral peduncle, internal capsule, corona radiata, superior and inferior longitudinal fasciculus, anterior and posterior thalamic radiation, external capsule, and superior cerebellar peduncle [89–91].

#### No-Sex Studies

No-sex DTI studies (*n* = 2) reported on the impact of exercise intervention on diffusivity parameters [42] and structural and functional connectivity parameters that best distinguished abdominal from non-abdominal obesity, indicating the importance of the frontoparietal and executive control networks [92].

### Evolution of the Treatment of Sex During the Study Period

We evaluated the proportion of studies that considered sex as an important biological variable in the analyses (direct-sex and single/within-sex studies) published in early (2010–2015) and later (after 2015) portions of the study period. Ten (12.8%) of 78 studies published in the early study period and 16 (13.2%) of 121 studies published in the later study period considered sex as an important variable. Accordingly, the majority of studies that considered sex as an important variable were published in the late study period (16/26; 61.5%).

### Keyword Co-occurrence Network

**Fig. 3:**
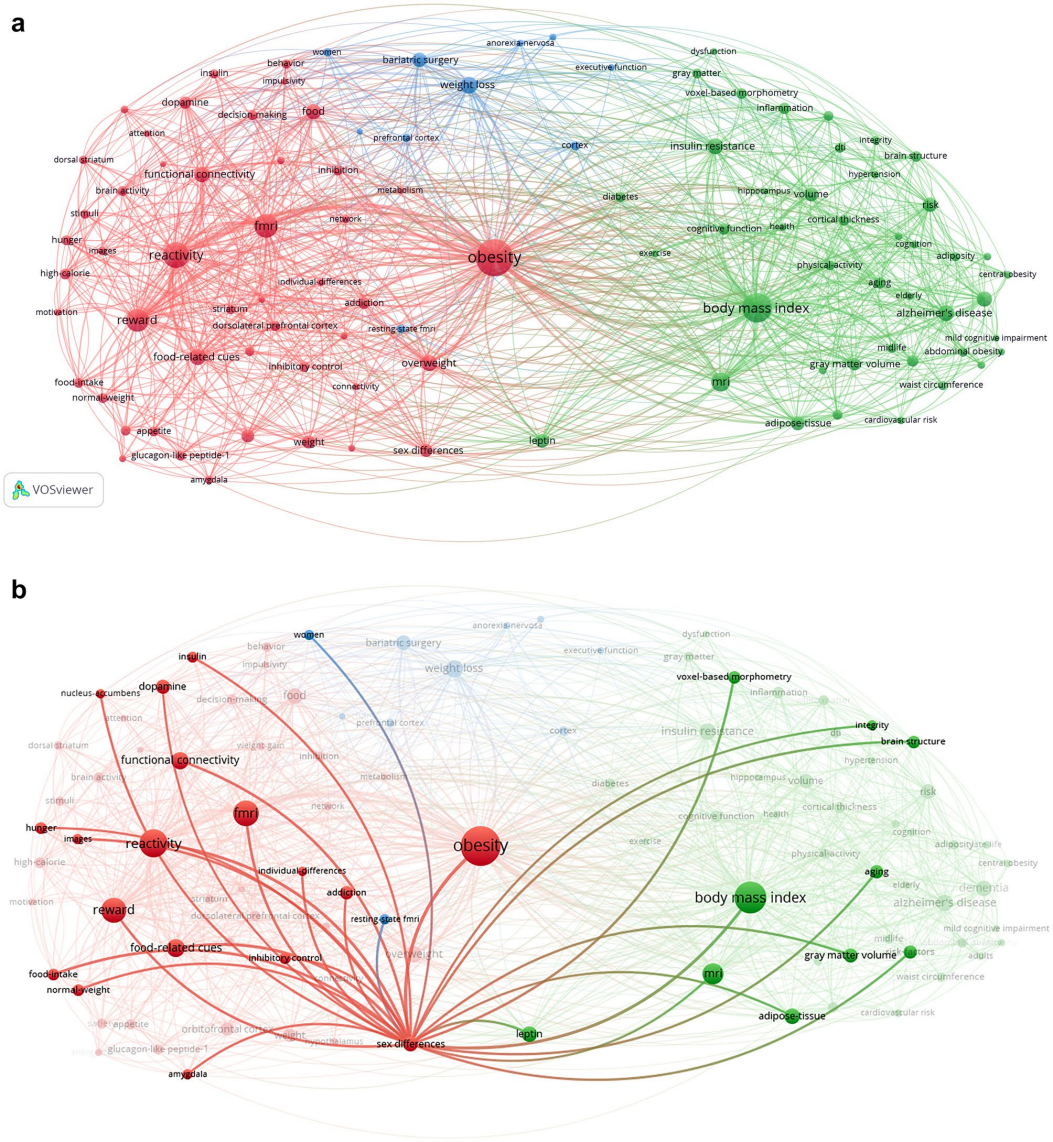
Results of the keyword co-occurrence network analysis. Clusters of the top 100 keywords are indicated by different colors in the network visualization, including a reward/reactivity cluster in red, disease-state cluster in green, and weight-loss intervention cluster in blue. (**a**) The entire network is shown. (**b**) Keywords frequently co-occurring with the keyword, sex differences, are highlighted

In the keyword co-occurrence network analysis of neuroimaging articles on adult obesity from 2010, three main clusters were identified: a reward/reactivity cluster, including keywords related to the extended reward system (e.g., striatum, dorsolateral PFC, amygdala) and reward-related functions (e.g., impulsivity, inhibitory control, decision-making), and brain reactivity to food-related cues and food intake; a disease-state cluster (e.g., dementia, insulin resistance, and Alzheimer’s disease); and a weight-loss intervention cluster (Fig. 3a). Although the keyword, sex differences, was included in, and well-connected with, the reward/reactivity cluster, it also had numerous links to the disease-state cluster, but did not show links with prominent intervention-related nodes (e.g. “weight loss,” “bariatric surgery”) (Fig. 3b).

## Discussion

In this review, we categorized adult obesity neuroimaging studies according to the treatment of sex in the analyses and found that only ~ 13% of the studies treated sex as an important variable. There was a general lack of studies that examined sex differences, or at least presented disaggregated data, which could be useful in developing sex-specific biomarkers and interventions. Only 10 studies (5%) directly compared the sexes, and 16 (8%) limited the analysis to a single sex or analyzed the sexes separately, allowing qualitative comparisons, which may be a first step in understanding the impact of sex. This is in stark contrast to the 120 studies (60%) that controlled for sex and 53 studies (27%) that did not consider sex in the analysis, disallowing any comparisons. Moreover, the few single-sex studies were disproportionately focused on women rather than on men. This reveals the need for more research on sex differences and men in the field of obesity neuroimaging. In order to better understand and treat obesity, differences in obesity effects on the brain between the two sexes need to be elucidated, especially as the present review indicates that such differences do exist.

### General Trends in Obesity Neuroimaging Findings According to Sex

The literature to date indicates that obesity is associated with numerous alterations in brain structure and responses to food-related stimuli; however, the extent and predominate regions involved differ between men and women with obesity, raising concerns regarding conclusions drawn from sex-adjusted and no-sex studies, as ignoring sex or adjusting for sex can mask important differences. Direct-sex studies generally showed more robust morphological alterations in subcortical and emotion-related regions in men [7–13] and more robust, widespread alterations in structural connectivity in women with obesity/high BMI/etc. [10, 87]. Additionally, women with obesity generally expressed increased reactivity to food-related cues in emotion-related regions, while men with obesity generally expressed increased reactivity to food-related cues in motor-related regions; this was especially true under fasting conditions [55–60].

### Major Knowledge Gaps in the Field

Investigations of sex effects were especially lacking in intervention studies. This was clearly apparent in the keyword co-occurrence, as the keyword, sex differences, did not show any links with intervention-related keywords, such as bariatric surgery and weight loss. The keyword co-occurrence analysis revealed other major areas of research with limited consideration of sex differences, which included disease states such as dementia, Alzheimer’s disease, and insulin resistance. Many of the no-sex studies focused on the impact of various interventions on brain measures in the general obese population and in specific patient populations. Because sex was ignored, its relative contribution to differences in the neural response to treatment is unclear. However, non-imaging studies suggest sex differences in obesity intervention effectiveness [93], and the direct-sex studies in the current review suggest sex differences in reward-related and emotional-regulation region alterations under obese conditions. This suggests that obesity interventions should be tailored according to sex, as addressing specific neural alterations should enhance intervention efficacy. If more is known about how obesity differs between the two sexes, treatment for obesity can be optimized for both men and women; hence, there is need for more studies that use neuroimaging tools to explore sex differences and sex-specific obesity effects.

### Limitations of This Review and the Field in General

Some of the studies reporting “sex-based” or “sex-specific” effects did not directly compare the sexes; rather, the researchers reported obesity-related effects in men and women considered separately. This approach (testing within each sex separately) can inflate the probability of concluding that a sex-specific effect is present compared to that when directly comparing the sexes [94]. In the present review, we coded such studies as single/within-sex, even though both sexes were included, as a direct comparison was lacking. This practice of testing within each sex separately may be unavoidable in typical small-sample neuroimaging studies that are underpowered for a sex interaction, but wish to provide information regarding potential sex effects. Many researchers have concerns about how to best conduct statistical analyses to assess the impact of sex in their dataset; a recent article provides an excellent conceptual guide [95•]. Additionally, partial least squares is a multivariate regression technique that is considered more sensitive than traditional neuroimaging analytic approach; as such, it may be better able to uncover sex differences [96]. Moreover, a focus on effect sizes, beyond *p*-values [95•, 97••], would help in understanding the scope of sex differences in the neural correlates of obesity and response to its treatment.

### Concluding Remarks on Future Directions

The earliest articles covered in this review were published nearly a decade after the Institute of Medicine reported on the importance of considering sex in the exploration of the biological contributions to human health in 2001 [98]. Despite this, only ~ 13% of the studies reviewed treated sex as an important biological variable. However, the National Health Institutes first announced their intention to consider sex as a biological variable and established such policies in 2015/2016 (i.e., around the midpoint of the period covered by this review) to improve research rigor [99]. Additionally, journals have increasingly incorporated policies on the reporting of sex effects in their guidelines in recent years [100•]. Thus, additional pressure to consider sex as an important variable was in place during the later portion of the current review period. Accordingly, the majority of direct-sex and single/within-sex studies were published in later portion of the study period, after 2015 (16/26; 61.5%); unfortunately, this only slightly raised the proportion of direct-sex and single/within-sex studies from 12.8% in 2010–2015 to 13.2% since 2016. However, we hope that this trend will accelerate, enabling such studies to become the majority during the 2020s, filling in the abovementioned knowledge gaps, and improving our understanding of the neural correlates of obesity and response to its treatment in men and women, to aid in the optimization of treatment strategies. In the meantime, it is important to realize that a large proportion of the obesity neuroimaging literature informing future research has ignored the importance of sex.

Supplementary Materials: The full bibliography of reviewed articles according to the treatment of sex in the analysis.

## Supplementary Information

Below is the link to the electronic supplementary material.

Supplementary file1 (DOCX 34 KB)
