# Supplementary material for: Neuroimaging Investigations of Obesity: a Review of the Treatment of Sex from 2010
Source: Curr Obes Rep. 2023 Mar 18;12(2):163–74. doi: 10.1007/s13679-023-00498-0 (PMC10250271; doi:10.1007/s13679-023-00498-0)
Supplement: Supplementary file 1 — Supplementary file1 (DOCX 34 KB) [file 13679_2023_498_MOESM1_ESM.docx]

Full Bibliography of Reviewed Articles

Direct sex

1. Atalayer, D., et al., Sexually dimorphic functional connectivity in response to high vs. low energy-dense food cues in obese humans: an fMRI study. Neuroimage, 2014. 100: p. 405-13.

2. Dekkers, I.A., P.R. Jansen, and H.J. Lamb, Obesity, Brain Volume, and White Matter Microstructure at MRI: A Cross-sectional UK Biobank Study. Radiology, 2019. 291(3): p. 763-771.

3. Geliebter, A., et al., Sex-based fMRI differences in obese humans in response to high vs. low energy food cues. Behav Brain Res, 2013. 243: p. 91-6.

4. Gupta, A., et al., Sex differences in the influence of body mass index on anatomical architecture of brain networks. International Journal of Obesity, 2017. 41(8): p. 1185-1195.

5. Gupta, A., et al., Sex Commonalities and Differences in Obesity-Related Alterations in Intrinsic Brain Activity and Connectivity. Obesity (Silver Spring), 2018. 26(2): p. 340-350.

6. Hempel, R., R. Onopa, and A. Convit, Type 2 diabetes affects hippocampus volume differentially in men and women. Diabetes Metab Res Rev, 2012. 28(1): p. 76-83.

7. Huang, Y., et al., Interaction Effect of Sex and Body Mass Index on Gray Matter Volume. Front Hum Neurosci, 2019. 13: p. 360.

8. Legget, K.T., et al., Greater Reward-Related Neuronal Response to Hedonic Foods in Women Compared with Men. Obesity (Silver Spring), 2018. 26(2): p. 362-367.

9. Sayer, R.D., et al., Reproducibility Assessment of Brain Responses to Visual Food Stimuli in Adults with Overweight and Obesity. Obesity, 2016. 24(10): p. 2057-2063.

10. Zerbini, C., et al., Shaping consumption propensity through the emotional response evoked by nutritional labels: Evidence from an fMRI study. Food research international (Ottawa, Ont.), 2019. 125: p. 108547.

Single/within sex

1. Arnoldussen, I.A.C., et al., Adiposity is related to cerebrovascular and brain volumetry outcomes in the RUN DMC study. Neurology, 2019. 93(9): p. E864-E878.

2. Coveleskie, K., et al., Altered functional connectivity within the central reward network in overweight and obese women. Nutrition & Diabetes, 2015. 5: p. 7.

3. Fehrenbach, U., et al., Obesity and pituitary gland volume – a correlation study using three-dimensional magnetic resonance imaging. Neuroradiology Journal, 2020. 33(5): p. 400-409.

4. Frankort, A., et al., Reward activity in satiated overweight women is decreased during unbiased viewing but increased when imagining taste: An event-related fMRI study. International Journal of Obesity, 2012. 36(5): p. 627-637.

5. Gobbi, S., et al., Reduced Neural Satiety Responses in Women Affected by Obesity. Neuroscience, 2020. 447: p. 94-112.

6. Hayakawa, Y.K., et al., The relationship of waist circumference and body mass index to grey matter volume in community dwelling adults with mild obesity. Obesity Science & Practice, 2018. 4(1): p. 97-105.

7. Hogenkamp, P.S., et al., Higher resting-state activity in reward-related brain circuits in obese versus normal-weight females independent of food intake. International Journal of Obesity, 2016. 40(11): p. 1687-1692.

8. Kerem, L., et al., Oxytocin reduces the functional connectivity between brain regions involved in eating behavior in men with overweight and obesity. International Journal of Obesity, 2020. 44(5): p. 980-989.

9. Killgore, W.D.S., et al., Cortico-limbic responsiveness to high-calorie food images predicts weight status among women. International Journal of Obesity, 2013. 37(11): p. 1435-1442.

10. Kim, H., et al., Association between body mass index and cortical thickness: among elderly cognitively normal men and women. Int Psychogeriatr, 2015. 27(1): p. 121-30.

11. Kim, H.J., et al., Association of Body Fat Percentage and Waist-hip Ratio With Brain Cortical Thickness: A Study Among 1777 Cognitively Normal Subjects. Alzheimer Dis Assoc Disord, 2015. 29(4): p. 279-86.

12. Mueller, K., et al., Sex-dependent influences of obesity on cerebral white matter investigated by diffusion-tensor imaging. PLoS One, 2011. 6(4): p. e18544.

13. Osadchiy, V., et al., History of early life adversity is associated with increased food addiction and sex-specific alterations in reward network connectivity in obesity. Obes Sci Pract, 2019. 5(5): p. 416-436.

14. Song, L.L.T., et al., Smaller size of high metabolic rate organs explains lower resting energy expenditure in Asian-Indian Than Chinese men. International Journal of Obesity, 2016. 40(4): p. 633-638.

15. Walther, K., et al., Structural Brain Differences and Cognitive Functioning Related to Body Mass Index in Older Females. Human Brain Mapping, 2010. 31(7): p. 1052-1064.

16. Zsido, R.G., et al., Association of Estradiol and Visceral Fat With Structural Brain Networks and Memory Performance in Adults. Jama Network Open, 2019. 2(6): p. 15.

Sex Adjusted

1. Albanese, E., et al., Overweight and Obesity in Midlife and Brain Structure and Dementia 26 Years Later: The AGES-Reykjavik Study. Am J Epidemiol, 2015. 181(9): p. 672-9.

2. Allen, B., et al., Higher Blood Pressure Partially Links Greater Adiposity to Reduced Brain White Matter Integrity. American Journal of Hypertension, 2016. 29(9): p. 1029-1037.

3. Ambikairajah, A., et al., Longitudinal Changes in Fat Mass and the Hippocampus. Obesity, 2020. 28(7): p. 1263-1269.

4. Armstrong, N.M., et al., Sex differences in brain aging and predictors of neurodegeneration in cognitively healthy older adults. Neurobiol Aging, 2019. 81: p. 146-156.

5. Avery, J.A., et al., Obesity is associated with altered mid-insula functional connectivity to limbic regions underlying appetitive responses to foods. Journal of Psychopharmacology, 2017. 31(11): p. 1475-1484.

6. Azor, A.M., et al., Increased brain age in adults with Prader-Willi syndrome. Neuroimage-Clinical, 2019. 21: p. 12.

7. Baboumian, S., et al., Functional Magnetic Resonance Imaging (fMRI) of Neural Responses to Visual and Auditory Food Stimuli Pre and Post Roux-en-Y Gastric Bypass (RYGB) and Sleeve Gastrectomy (SG). Neuroscience, 2019. 409: p. 290-298.

8. Bae, J.H., et al., Glucagon-like peptide-1 receptor agonist differentially affects brain activation in response to visual food cues in lean and obese individuals with type 2 diabetes mellitus. Diabetes and Metabolism Journal, 2019. 43.

9. Balodis, I.M., et al., Monetary reward processing in obese individuals with and without binge eating disorder. Biological Psychiatry, 2013. 73(9): p. 877-886.

10. Balodis, I.M., et al., Divergent neural substrates of inhibitory control in binge eating disorder relative to other manifestations of obesity. Obesity, 2013. 21(2): p. 367-377.

11. Beller, E., et al., Hepatic fat is superior to BMI, visceral and pancreatic fat as a potential risk biomarker for neurodegenerative disease. Eur Radiol, 2019. 29(12): p. 6662-6670.

12. Beyer, F., et al., A metabolic obesity profile is associated with decreased gray matter volume in cognitively healthy older adults. Frontiers in Aging Neuroscience, 2019. 10(JUL).

13. Binda, P., et al., Exenatide modulates visual cortex responses. Diabetes/Metabolism Research and Reviews, 2019. 35(6).

14. Birdsill, A.C., et al., Abdominal Obesity and White Matter Microstructure in Midlife. Human Brain Mapping, 2017. 38(7): p. 3337-3344.

15. Bolzenius, J.D., et al., Impact of body mass index on neuronal fiber bundle lengths among healthy older adults. Brain Imaging and Behavior, 2013. 7(3): p. 300-306.

16. Bolzenius, J.D., et al., Brain structure and cognitive correlates of body mass index in healthy older adults. Behavioural Brain Research, 2015. 278: p. 342-347.

17. Bond, D.J., et al., The association of elevated body mass index with reduced brain volumes in first-episode mania. Biological Psychiatry, 2011. 70(4): p. 381-387.

18. Boyle, C.P., et al., Physical activity, body mass index, and brain atrophy in Alzheimer's disease. Neurobiology of Aging, 2015. 36: p. S194-S202.

19. Brooks, S.J., et al., Late-life obesity is associated with smaller global and regional gray matter volumes: A voxel-based morphometric study. International Journal of Obesity, 2013. 37(2): p. 230-236.

20. Burdette, J.H., et al., Functional Brain Networks: Unique Patterns with Hedonic Appetite and Confidence to Resist Eating in Older Adults with Obesity. Obesity, 2020. 28(12): p. 2379-2388.

21. Castro, M.G., et al., Fitness, insulin sensitivity, and frontal lobe integrity in adults with overweight and obesity. Obesity, 2016. 24(6): p. 1283-1289.

22. Caunca, M.R., et al., Measures of obesity are associated with MRI markers of brain aging The Northern Manhattan Study. Neurology, 2019. 93(8): p. E791-E803.

23. Caunca, M.R., et al., Measures of Adiposity and Alzheimer's Disease-Related MRI Markers: The Northern Manhattan Study. Journal of Alzheimer's Disease, 2019. 70(4): p. 995-1004.

24. Cazettes, F., et al., Obesity-mediated inflammation may damage the brain circuit that regulates food intake. Brain Research, 2011. 1373: p. 101-109.

25. Cheke, L.G., et al., Obesity and insulin resistance are associated with reduced activity in core memory regions of the brain. Neuropsychologia, 2017. 96: p. 137-149.

26. Cherbuin, N., et al., Being overweight is associated with hippocampal atrophy: The PATH Through Life Study. International Journal of Obesity, 2015. 39(10): p. 1509-1514.

27. Climie, R.E.D., et al., Abdominal Obesity and Brain Atrophy in Type 2 Diabetes Mellitus. Plos One, 2015. 10(11): p. 11.

28. Cohen, J.I., F. Cazettes, and A. Convit, Abnormal cholesterol is associated with prefrontal white matter abnormalities among obese adults: A diffusion tensor imaging study. Neuroradiology Journal, 2011. 24(6): p. 854-861.

29. Contreras-Rodriguez, O., et al., Visceral adiposity and insular networks: associations with food craving. International Journal of Obesity, 2019. 43(3): p. 503-511.

30. Contreras-Rodríguez, O., et al., Ventral and Dorsal Striatum Networks in Obesity: Link to Food Craving and Weight Gain. Biological Psychiatry, 2017. 81(9): p. 789-796.

31. Contreras-Rodriguez, O., et al., Neural-based valuation of functional foods among lean and obese individuals. Nutrition Research, 2020. 78: p. 27-35.

32. Contreras-Rodríguez, O., et al., Altered cross-talk between the hypothalamus and non-homeostatic regions linked to obesity and difficulty to lose weight. Scientific reports, 2017. 7(1): p. 9951.

33. De Groot, C., et al., Association of the fat mass and obesity-associated gene risk allele, rs9939609A, and reward-related brain structures. Obesity, 2015. 23(10): p. 2118-2122.

34. Dearborn, J.L., et al., Obesity, Insulin Resistance, and Incident Small Vessel Disease on Magnetic Resonance Imaging: Atherosclerosis Risk in Communities Study. Stroke, 2015. 46(11): p. 3131-3136.

35. Debette, S., et al., Visceral Fat Is Associated with Lower Brain Volume in Healthy Middle-Aged Adults. Annals of Neurology, 2010. 68(2): p. 136-144.

36. Debette, S., et al., Abdominal obesity and lower gray matter volume: a Mendelian randomization study. Neurobiology of Aging, 2014. 35(2): p. 378-386.

37. Demos McDermott, K.E., et al., Effects of Cognitive Strategies on Neural Food Cue Reactivity in Adults with Overweight/Obesity. Obesity, 2019. 27(10): p. 1577-1583.

38. Driscoll, I., et al., Midlife obesity and trajectories of brain volume changes in older adults. Human Brain Mapping, 2012. 33(9): p. 2204-2210.

39. Drummen, M., et al., Insulin resistance, weight, and behavioral variables as determinants of brain reactivity to food cues: a Prevention of Diabetes through Lifestyle Intervention and Population Studies in Europe and around the World - a PREVIEW study. Am J Clin Nutr, 2019. 109(2): p. 315-321.

40. Drummen, M., et al., Reductions in body weight and insulin resistance are not associated with changes in grey matter volume or cortical thickness during the PREVIEW study. Journal of the Neurological Sciences, 2019. 403: p. 106-111.

41. Eckstrand, K.L., et al., An insulin resistance associated neural correlate of impulsivity in type 2 diabetes mellitus. PLoS One, 2017. 12(12): p. e0189113.

42. Eldor, R., et al., Discordance between central (Brain) and pancreatic action of exenatide in lean and obese subjects. Diabetes Care, 2016. 39(10): p. 1804-1810.

43. Farr, O.M. and C.S. Mantzoros, Obese individuals with more components of the metabolic syndrome and/or prediabetes demonstrate decreased activation of reward-related brain centers in response to food cues in both the fed and fasting states: A preliminary fMRI study. International Journal of Obesity, 2017. 41(3): p. 471-474.

44. Farr, O.M. and C.S. Mantzoros, Obese individuals with type 2 diabetes demonstrate decreased activation of the salience-related insula and increased activation of the emotion/salience-related amygdala to visual food cues compared to non-obese individuals with diabetes: A preliminary study. Diabetes, Obesity and Metabolism, 2018. 20(10): p. 2500-2503.

45. Fernandez-Real, J.M., et al., Gut Microbiota Interacts With Brain Microstructure and Function. Journal of Clinical Endocrinology & Metabolism, 2015. 100(12): p. 4505-4513.

46. Filbey, F.M. and U.S. Yezhuvath, A multimodal study of impulsivity and body weight: Integrating behavioral, cognitive, and neuroimaging approaches. Obesity, 2017. 25(1): p. 147-154.

47. Gonzales, M.M., et al., Central adiposity and the functional magnetic resonance imaging response to cognitive challenge. International Journal of Obesity, 2014. 38(9): p. 1193-1199.

48. Grosshans, M., et al., Oleoylethanolamide and human neural responses to food stimuli in obesity. JAMA Psychiatry, 2014. 71(11): p. 1254-1261.

49. Grosshans, M., et al., Association of Leptin With Food Cue-Induced Activation in Human Reward Pathways. Archives of General Psychiatry, 2012. 69(5): p. 529-537.

50. Grosshans, M., et al., The association of pineal gland volume and body mass in obese and normal weight individuals: A pilot study. Psychiatria Danubina, 2016. 28(3): p. 220-224.

51. Gupta, A., et al., Patterns of brain structural connectivity differentiate normal weight from overweight subjects. Neuroimage-Clinical, 2015. 7: p. 506-517.

52. Hamer, M. and G.D. Batty, Association of body mass index and waist-to-hip ratio with brain structure: UK Biobank study. Neurology, 2019. 92(6): p. e594-e600.

53. Hassenstab, J.J., et al., Cortical thickness of the cognitive control network in obesity and successful weight loss maintenance: A preliminary MRI study. Psychiatry Research - Neuroimaging, 2012. 202(1): p. 77-79.

54. Heni, M., et al., Interaction between the obesity-risk gene FTO and the dopamine D2 receptor gene ANKK1/TaqIA on insulin sensitivity. Diabetologia, 2016. 59(12): p. 2622-2631.

55. Heni, M., et al., Differential effect of glucose ingestion on the neural processing of food stimuli in lean and overweight adults. Human Brain Mapping, 2014. 35(3): p. 918-928.

56. Heni, M., et al., Variation in the obesity risk gene FTO determines the postprandial cerebral processing of food stimuli in the prefrontal cortex. Molecular Metabolism, 2014. 3(2): p. 109-113.

57. Hidese, S., et al., Association of obesity with cognitive function and brain structure in patients with major depressive disorder. Journal of Affective Disorders, 2018. 225: p. 188-194.

58. Ho, A., J. Kennedy, and A. Dimitropoulos, Neural Correlates to Food-Related Behavior in Normal-Weight and Overweight/Obese Participants. PLoS ONE, 2012. 7(9).

59. Ho, M.C., et al., Neural correlates of executive functions in patients with obesity. Peerj, 2018. 6: p. 18.

60. Holsen, L.M., et al., Neural predictors of 12-month weight loss outcomes following bariatric surgery. International Journal of Obesity, 2018. 42(4): p. 785-793.

61. Honea, R.A., et al., Voxel-based morphometry reveals brain gray matter volume changes in successful dieters. Obesity, 2016. 24(9): p. 1842-1848.

62. Hsu, C.L., et al., Elevated body mass index and maintenance of cognitive function in late life: Exploring underlying neural mechanisms. Frontiers in Aging Neuroscience, 2015. 7(JUL).

63. Hsu, F.C., et al., Adiposity is inversely associated with hippocampal volume in African Americans and European Americans with diabetes. Journal of Diabetes and its Complications, 2016. 30(8): p. 1506-1512.

64. Hu, Y., et al., Laparoscopic sleeve gastrectomy improves brain connectivity in obese patients. Journal of Neurology, 2020. 267(7): p. 1931-1940.

65. Jacobson, A., et al., Differential effects of BMI on brain response to odor in olfactory, reward and memory regions: Evidence from fMRI. Nutrients, 2019. 11(4).

66. Janowitz, D., et al., Association between waist circumference and gray matter volume in 2344 individuals from two adult community-based samples. Neuroimage, 2015. 122: p. 149-157.

67. Janssen, L.K., et al., Loss of lateral prefrontal cortex control in food-directed attention and goal-directed food choice in obesity. NeuroImage, 2017. 146: p. 148-156.

68. Jastreboff, A.M., et al., Neural correlates of stress- and food cue-induced food craving in obesity: Association with insulin levels. Diabetes Care, 2013. 36(2): p. 394-402.

69. Kim, K.W., et al., Visceral obesity is associated with white matter hyperintensity and lacunar infarct. International Journal of Obesity, 2017. 41(5): p. 683-688.

70. Kotkowski, E., et al., A neural signature of metabolic syndrome. Human Brain Mapping, 2019. 40(12): p. 3575-3588.

71. Kullmann, S., et al., Specific white matter tissue microstructure changes associated with obesity. NeuroImage, 2016. 125: p. 36-44.

72. Kullmann, S., et al., Functional network connectivity underlying food processing: Disturbed salience and visual processing in overweight and obese adults. Cerebral Cortex, 2013. 23(5): p. 1247-1256.

73. Lampe, L., et al., Visceral obesity relates to deep white matter hyperintensities via inflammation. Annals of Neurology, 2019. 85(2): p. 194-203.

74. Le, T.M., et al., The interrelationship of body mass index with gray matter volume and resting-state functional connectivity of the hypothalamus. International Journal of Obesity, 2020. 44(5): p. 1097-1107.

75. Legget, K., et al., Altered Network Connectivity in Individuals Prone to Obesity. Biological Psychiatry, 2019. 85(10): p. S238.

76. Li, G., et al., Resting activity of the hippocampus and amygdala in obese individuals predicts their response to food cues. Addiction Biology, 2020.

77. Li, G., et al., Reduced plasma ghrelin concentrations are associated with decreased brain reactivity to food cues after laparoscopic sleeve gastrectomy. Psychoneuroendocrinology, 2019. 100: p. 229-236.

78. Li, G., et al., Bariatric surgery in obese patients reduced resting connectivity of brain regions involved with self-referential processing. Human Brain Mapping, 2018. 39(12): p. 4755-4765.

79. Liu, L., et al., Structural changes in brain regions involved in executive-control and self-referential processing after sleeve gastrectomy in obese patients. Brain Imaging and Behavior, 2019. 13(3): p. 830-840.

80. Lopez, R.B., T.F. Heatherton, and D.D. Wagner, Media multitasking is associated with higher risk for obesity and increased responsiveness to rewarding food stimuli. Brain Imaging and Behavior, 2020. 14(4): p. 1050-1061.

81. Lou, B., et al., Reduced right frontal fractional anisotropy correlated with early elevated plasma LDL levels in obese young adults. PLoS ONE, 2014. 9(10).

82. Marks, B.L., et al., Aerobic fitness and obesity: relationship to cerebral white matter integrity in the brain of active and sedentary older adults. British Journal of Sports Medicine, 2011. 45(15): p. 1208-1215.

83. Mathar, D., et al., Is it Worth the Effort? Novel Insights into Obesity-Associated Alterations in Cost-Benefit Decision-Making. Frontiers in Behavioral Neuroscience, 2016. 9: p. 13.

84. Maurer, L., et al., Interaction of circulating GLP-1 and the response of the dorsolateral prefrontal cortex to food-cues predicts body weight development. Molecular Metabolism, 2019. 29: p. 136-144.

85. Michaud, A., et al., Neuroanatomical changes in white and grey matter after sleeve gastrectomy. Neuroimage, 2020. 213: p. 9.

86. Pegueroles, J., et al., Obesity and Alzheimer's disease, does the obesity paradox really exist? A magnetic resonance imaging study. Oncotarget, 2018. 9(78): p. 34691-34698.

87. Rajagopalan, P., et al., Fat-mass-related hormone, plasma leptin, predicts brain volumes in the elderly. Neuroreport, 2013. 24(2): p. 58-62.

88. Raschpichler, M., et al., Abdominal fat distribution and its relationship to brain changes: The differential effects of age on cerebellar structure and function: A cross-sectional, exploratory study. BMJ Open, 2013. 3(1).

89. Reckziegel, R., et al., Obesity and brain integrity in schizophrenia and bipolar disorder: Divergent patterns of white matter microstructure damage in a transdiagnostic approach. Schizophrenia Bulletin, 2018. 44: p. S288-S289.

90. Reyes, S., et al., Assessing cognitive control and the reward system in overweight young adults using sensitivity to incentives and white matter integrity. PLoS ONE, 2020. 15(6).

91. Romer, A.L., et al., Dopamine genetic risk is related to food addiction and body mass through reduced reward-related ventral striatum activity. Appetite, 2019. 133: p. 24-31.

92. Sala, M., et al., Liver fat assessed with CT relates to MRI markers of incipient brain injury in middle-aged to elderly overweight persons. American Journal of Roentgenology, 2016. 206(5): p. 1087-1092.

93. Schall, M., et al., Increasing body mass index in an elderly cohort: Effects on the quantitative MR parameters of the brain. Journal of Magnetic Resonance Imaging, 2020. 51(2): p. 514-523.

94. Scholtz, S., et al., Obese patients after gastric bypass surgery have lower brain-hedonic responses to food than after gastric banding. Gut, 2014. 63(6): p. 891-902.

95. Simon, J.J., et al., Impaired cross-talk between mesolimbic food reward processing and metabolic signaling predicts body mass index. Frontiers in Behavioral Neuroscience, 2014. 8(OCT): p. 1-10.

96. Smucny, J., et al., Brain structure predicts risk for obesity. Appetite, 2012. 59(3): p. 859-65.

97. Spieker, E.A., et al., Shared genetic variance between obesity and white matter integrity in Mexican Americans. Frontiers in Genetics, 2015. 5(FEB).

98. Stice, E., K. Burger, and S. Yokum, Caloric deprivation increases responsivity of attention and reward brain regions to intake, anticipated intake, and images of palatable foods. NeuroImage, 2013. 67: p. 322-330.

99. Stice, E., et al., A pilot randomized trial of a cognitive reappraisal obesity prevention program. Physiology and Behavior, 2015. 138: p. 124-132.

100. Takahashi, H., et al., Effects of sports participation on psychiatric symptoms and brain activations during sports observation in schizophrenia. Translational Psychiatry, 2012. 2: p. 6.

101. Thomas, K., et al., Higher body mass index is linked to altered hypothalamic microstructure. Scientific Reports, 2019. 9: p. 11.

102. van Bloemendaal, L., et al., Alterations in white matter volume and integrity in obesity and type 2 diabetes. Metabolic Brain Disease, 2016. 31(3): p. 621-629.

103. Verdejo-Román, J., et al., Brain reward system's alterations in response to food and monetary stimuli in overweight and obese individuals. Human Brain Mapping, 2017. 38(2): p. 666-677.

104. Vergoossen, L.W.M., et al., Association of physical activity and sedentary time with structural brain networks-The Maastricht Study. Geroscience, 2021. 43: p. 14.

105. Verhaar, B.J.H., et al., Nutritional status and structural brain changes in Alzheimer's disease: The NUDAD project. Alzheimer's and Dementia: Diagnosis, Assessment and Disease Monitoring, 2020. 12(1).

106. Verstynen, T.D., et al., Competing physiological pathways link individual differences in weight and abdominal adiposity to white matter microstructure. NeuroImage, 2013. 79: p. 129-137.

107. Verstynen, T.D., et al., Increased body mass index is associated with a global and distributed decrease in white matter microstructural integrity. Psychosomatic Medicine, 2012. 74(7): p. 682-690.

108. Virecoulon Giudici, K., et al., Body Weight Variation Patterns as Predictors of Cognitive Decline over a 5 Year Follow-Up among Community-Dwelling Elderly (MAPT Study). Nutrients, 2019. 11(6).

109. Walhovd, K.B., et al., Blood markers of fatty acids and vitamin D, cardiovascular measures, body mass index, and physical activity relate to longitudinal cortical thinning in normal aging. Neurobiology of Aging, 2014. 35(5): p. 1055-1064.

110. Wang, H., et al., Brain Structural Differences between Normal and Obese Adults and their Links with Lack of Perseverance, Negative Urgency, and Sensation Seeking. Scientific reports, 2017. 7: p. 40595.

111. Weise, C.M., T. Bachmann, and B. Pleger, Brain structural differences in monozygotic twins discordant for body mass index. NeuroImage, 2019. 201.

112. Weise, C.M., et al., The obese brain as a heritable phenotype: A combined morphometry and twin study. International Journal of Obesity, 2017. 41(3): p. 458-466.

113. Weygandt, M., et al., The role of neural impulse control mechanisms for dietary success in obesity. NeuroImage, 2013. 83: p. 669-678.

114. Weygandt, M., et al., Impulse control in the dorsolateral prefrontal cortex counteracts post-diet weight regain in obesity. NeuroImage, 2015. 109: p. 318-327.

115. Weygandt, M., et al., Interactions between neural decision-making circuits predict long-term dietary treatment success in obesity. NeuroImage, 2019. 184: p. 520-534.

116. Windham, B.G., et al., Associations of Brain Structure With Adiposity and Changes in Adiposity in a Middle-Aged and Older Biracial Population. The journals of gerontology. Series A, Biological sciences and medical sciences, 2017. 72(6): p. 825-831.

117. Zade, D., et al., Apolipoprotein Epsilon 4 Allele Modifies Waist-to-Hip Ratio Effects on Cognition and Brain Structure. Journal of Stroke & Cerebrovascular Diseases, 2013. 22(2): p. 119-125.

118. Zhang, R., et al., White matter microstructural variability mediates the relation between obesity and cognition in healthy adults. NeuroImage, 2018. 172: p. 239-249.

119. Zhang, Y., et al., Recovery of brain structural abnormalities in morbidly obese patients after bariatric surgery. International Journal of Obesity, 2016. 40(10): p. 1558-1565.

120. Zhang, Z., et al., Olfactory Dysfunction Mediates Adiposity in Cognitive Impairment of Type 2 Diabetes: Insights From Clinical and Functional Neuroimaging Studies. Diabetes Care, 2019. 42(7): p. 1274-1283.

No sex

1. Babbs, R.K., et al., Decreased caudate response to milkshake is associated with higher body mass index and greater impulsivity. Physiology and Behavior, 2013. 121: p. 103-111.

2. Bohon, C., L.C. Garcia, and J.M. Morton, Changes in Cerebral Cortical Thickness Related to Weight Loss Following Bariatric Surgery. Obesity Surgery, 2018. 28(8): p. 2578-2582.

3. Bohon, C. and A. Geliebter, Change in brain volume and cortical thickness after behavioral and surgical weight loss intervention. NeuroImage: Clinical, 2019. 21.

4. Bruce, A.S., et al., A comparison of functional brain changes associated with surgical versus behavioral weight loss. Obesity, 2014. 22(2): p. 337-343.

5. Cerit, H., et al., Resting-State Brain Connectivity Predicts Weight Loss and Cognitive Control of Eating Behavior After Vertical Sleeve Gastrectomy. Obesity, 2019. 27(11): p. 1846-1855.

6. Cornier, M.A., et al., The effects of exercise on the neuronal response to food cues. Physiol Behav, 2012. 105(4): p. 1028-34.

7. Deckersbach, T., et al., Pilot randomized trial demonstrating reversal of obesity-related abnormalities in reward system responsivity to food cues with a behavioral intervention. Nutrition and Diabetes, 2014. 4(9).

8. Drummen, M., et al., Associations of brain reactivity to food cues with weight loss, protein intake and dietary restraint during the PREVIEW intervention. Nutrients, 2018. 10(11).

9. Espeland, M.A., et al., Brain and white matter hyperintensity volumes after 10 years of random assignment to lifestyle intervention. Diabetes Care, 2016. 39(5): p. 764-771.

10. Farr, O.M., et al., Lorcaserin administration decreases activation of brain centers in response to food cues and these emotion- and salience-related changes correlate with weight loss effects: A 4-week-long randomized, placebo-controlled, double-blind clinical trial. Diabetes, 2016. 65(10): p. 2943-2953.

11. Farr, O.M., et al., Longer-term liraglutide administration at the highest dose approved for obesity increases reward-related orbitofrontal cortex activation in response to food cues: Implications for plateauing weight loss in response to anti-obesity therapies. Diabetes, Obesity and Metabolism, 2019. 21(11): p. 2459-2464.

12. Fletcher, P.C., et al., Distinct modulatory effects of satiety and sibutramine on brain responses to food images in humans: A double dissociation across hypothalamus, amygdala, and ventral striatum. Journal of Neuroscience, 2010. 30(43): p. 14346-14355.

13. Goldman, R.L., et al., Executive control circuitry differentiates degree of success in weight loss following gastric-bypass surgery. Obesity, 2013. 21(11): p. 2189-2196.

14. Han, P., H. Chen, and T. Hummel, Brain Responses to Food Odors Associated With BMI Change at 2-Year Follow-Up. Frontiers in Human Neuroscience, 2020. 14.

15. Hege, M.A., et al., Working memory-related brain activity is associated with outcome of lifestyle intervention. Obesity, 2013. 21(12): p. 2488-2494.

16. Heni, M., et al., Dissociation of GLP-1 and insulin association with food processing in the brain: GLP-1 sensitivity despite insulin resistance in obese humans. Molecular Metabolism, 2015. 4(12): p. 971-976.

17. Hermann, P., et al., Efficacy of weight loss intervention can be predicted based on early alterations of fMRI food cue reactivity in the striatum. NeuroImage: Clinical, 2019. 23.

18. Hinkle, W., et al., Effects of Reduced Weight Maintenance and Leptin Repletion on Functional Connectivity of the Hypothalamus in Obese Humans. PLoS ONE, 2013. 8(3).

19. Ho, A.J., et al., A commonly carried allele of the obesity-related FTO gene is associated with reduced brain volume in the healthy elderly. Proceedings of the National Academy of Sciences of the United States of America, 2010. 107(18): p. 8404-8409.

20. Jastreboff, A.M., et al., Body mass index, metabolic factors, and striatal activation during stressful and neutral-relaxing states: An fMRI study. Neuropsychopharmacology, 2011. 36(3): p. 627-637.

21. Kahathuduwa, C.N., et al., Do scores on the Food Craving Inventory and Three-Factor Eating Questionnaire correlate with expected brain regions of interest in people with obesity? Physiol Behav, 2018. 188: p. 1-10.

22. Kahathuduwa, C.N., et al., Effects of 3-week total meal replacement vs. typical food-based diet on human brain functional magnetic resonance imaging food-cue reactivity and functional connectivity in people with obesity. Appetite, 2018. 120: p. 431-441.

23. Kim, S.H., et al., The effects of high-frequency repetitive transcranial magnetic stimulation on resting-state functional connectivity in obese adults. Diabetes, Obesity and Metabolism, 2019. 21(8): p. 1956-1966.

24. Kohl, S.H., et al., Real-time fMRI neurofeedback training to improve eating behavior by self-regulation of the dorsolateral prefrontal cortex: A randomized controlled trial in overweight and obese subjects. NeuroImage, 2019. 191: p. 596-609.

25. Lepping, R.J., et al., Resting-state brain connectivity after surgical and behavioral weight loss. Obesity, 2015. 23(7): p. 1422-1428.

26. Li, P., et al., Sleeve Gastrectomy Rescuing the Altered Functional Connectivity of Lateral but Not Medial Hypothalamus in Subjects with Obesity. Obesity Surgery, 2019.

27. Li, P.L., et al., Sleeve Gastrectomy Recovering Disordered Brain Function in Subjects with Obesity: a Longitudinal fMRI Study. Obesity Surgery, 2018. 28(8): p. 2421-2428.

28. Marques-Iturria, I., et al., Frontal cortical thinning and subcortical volume reductions in early adulthood obesity. Psychiatry Research-Neuroimaging, 2013. 214(2): p. 109-115.

29. McDermott, K.D., et al., Impact of Intensive Lifestyle Intervention on Neural Food Cue Reactivity: Action for Health in Diabetes Brain Ancillary Study. Obesity, 2019. 27(7): p. 1076-1084.

30. Merchant, J.S., et al., Neural Substrates of Food Valuation and Its Relationship With BMI and Healthy Eating in Higher BMI Individuals. Frontiers in Behavioral Neuroscience, 2020. 14.

31. Mueller, K., et al., Physical exercise in overweight to obese individuals induces metabolic-and neurotrophic-related structural brain plasticity. Frontiers in Human Neuroscience, 2015. 9(JULY).

32. Murdaugh, D.L., et al., FMRI reactivity to high-calorie food pictures predicts short- and long-term outcome in a weight-loss program. NeuroImage, 2012. 59(3): p. 2709-2721.

33. Nakamura, Y. and T. Ikuta, Caudate-Precuneus Functional Connectivity Is Associated with Obesity Preventive Eating Tendency. Brain Connectivity, 2017. 7(3): p. 211-217.

34. Neseliler, S., et al., Neurocognitive and Hormonal Correlates of Voluntary Weight Loss in Humans. Cell Metabolism, 2019. 29(1): p. 39-49.e4.

35. Ness, A., et al., Pre-surgical cortical activation to food pictures is associated with weight loss following bariatric surgery. Surgery for Obesity and Related Diseases, 2014. 10(6): p. 1188-1195.

36. Opel, N., et al., Enhanced Neural Responsiveness to Reward Associated With Obesity in the Absence of Food-Related Stimuli. Human Brain Mapping, 2015. 36(6): p. 2330-2337.

37. Park, B.Y., et al., Structural and Functional Brain Connectivity Changes Between People With Abdominal and Non-abdominal Obesity and Their Association With Behaviors of Eating Disorders. Frontiers in Neuroscience, 2018. 12: p. 13.

38. Prehn, K., et al., Bariatric surgery and brain health—a longitudinal observational study investigating the effect of surgery on cognitive function and gray matter volume. Nutrients, 2020. 12(1).

39. Rotenstein, L.S., et al., Effect of mineralocorticoid receptor blockade on hippocampal-dependent memory in adults with obesity. Obesity, 2015. 23(6): p. 1136-1142.

40. Rullmann, M., et al., Gastric-bypass surgery induced widespread neural plasticity of the obese human brain. NeuroImage, 2018. 172: p. 853-863.

41. Saindane, A.M., et al., Neuroimaging correlates of cognitive changes after bariatric surgery. Surgery for Obesity and Related Diseases, 2020. 16(1): p. 119-127.

42. Sayer, R.D., et al., Effects of Dietary Protein and Fiber at Breakfast on Appetite, ad Libitum Energy Intake at Lunch, and Neural Responses to Visual Food Stimuli in Overweight Adults. Nutrients, 2016. 8(1).

43. Sayer, R.D., et al., Consuming Almonds vs. Isoenergetic Baked Food Does Not Differentially Influence Postprandial Appetite or Neural Reward Responses to Visual Food Stimuli. Nutrients, 2017. 9(8).

44. Sun, X., et al., Perceptual and brain response to odors is associated with body mass index and postprandial total ghrelin reactivity to a meal. Chemical Senses, 2016. 41(3): p. 233-248.

45. Szabo-Reed, A.N., et al., Modeling interactions between brain function, diet adherence behaviors, and weight loss success. Obesity Science and Practice, 2020. 6(3): p. 282-292.

46. Ten Kulve, J.S., et al., Liraglutide reduces CNS activation in response to visual food cues only after short-term treatment in patients with type 2 diabetes. Diabetes Care, 2016. 39(2): p. 214-221.

47. Ten Kulve, J.S., et al., Endogenous GLP1 and GLP1 analogue alter CNS responses to palatable food consumption. Journal of Endocrinology, 2016. 229(1): p. 1-12.

48. Tuulari, J.J., et al., Bariatric Surgery Induces White and Grey Matter Density Recovery in the Morbidly Obese: A Voxel-Based Morphometric Study. Human Brain Mapping, 2016. 37(11): p. 3745-3756.

49. Van Bloemendaal, L., et al., GLP-1 receptor activation modulates appetite- and reward-related brain areas in humans. Diabetes, 2014. 63(12): p. 4186-4196.

50. Wang, Y., et al., Laparoscopic sleeve gastrectomy induces sustained changes in gray and white matter brain volumes and resting functional connectivity in obese patients. Surgery for Obesity and Related Diseases, 2020. 16(1): p. 1-9.

51. Wijngaarden, M.A., et al., Obesity is marked by distinct functional connectivity in brain networks involved in food reward and salience. Behavioural Brain Research, 2015. 287: p. 127-134.

52. Yamashiro, K., et al., Visceral fat accumulation is associated with cerebral small vessel disease. European Journal of Neurology, 2014. 21(4): p. 667-673.

53. Zhang, W., et al., Connectome-Based Prediction of Optimal Weight Loss Six Months After Bariatric Surgery. Cerebral cortex (New York, N.Y. : 1991), 2020.
